# Investigation of a relationship between serum concentrations of microRNA-122 and alanine aminotransferase activity in hospitalised cats

**DOI:** 10.1177/1098612X221100071

**Published:** 2022-06-15

**Authors:** Susan K Armstrong, Wilna Oosthuyzen, Adam G Gow, Silke Salavati Schmitz, James W Dear, Richard J Mellanby

**Affiliations:** 1 The Royal (Dick) School of Veterinary Studies and the Roslin Institute, The Hospital for Small Animals, University of Edinburgh, Edinburgh, UK; 2 Pharmacology, Toxicology and Therapeutics, Centre for Cardiovascular Science, University of Edinburgh, Edinburgh, UK; 3 The Roslin Institute, University of Edinburgh, Edinburgh, UK

**Keywords:** Hepatic disease, microRNA, biomarker, miR-122

## Abstract

**Objectives:**

Current blood tests to diagnose feline liver diseases are suboptimal. Serum concentrations of microRNA (miR)-122 have been shown in humans, dogs and rodents to be a sensitive and specific biomarker for liver injury. To explore the potential diagnostic utility of measuring serum concentrations of miR-122 in cats, miR-122 was measured in a cohort of ill, hospitalised cats with known serum alanine aminotransferase (ALT) activity.

**Methods:**

In this retrospective study, cats were grouped into those with an ALT activity within the reference interval (0–83 U/l; n = 38) and those with an abnormal ALT activity (>84 U/l; n = 25). Serum concentrations of miR-122 were measured by real-time quantitative PCR and the relationship between miR-122 and ALT was examined.

**Results:**

miR-122 was significantly higher in the group with high ALT activity than the ALT group, within normal reference limits (*P* <0.0004). There was also a moderately positive correlation between serum ALT activity and miR-122 concentrations (*P* <0.001; *r* = 0.52).

**Conclusions and relevance:**

Concentrations of miR-122 were reliably quantified in feline serum and were higher in a cohort of cats with increased ALT activity than in cats with normal ALT activity. This work highlights the potential diagnostic utility of miR-122 as a biomarker of liver damage in cats and encourages further investigation to determine the sensitivity and specificity of miR-122 as a biomarker of hepatocellular injury in this species.

## Introduction

Hepatic disease is an important cause of morbidity and mortality in cats.^1^ The diagnosis of hepatic disorders in cats can be challenging, primarily due to non-specific clinical signs. Initial screening for the presence of hepatic injury relies on the measurement of liver enzymes such as alanine aminotransferase (ALT) and aspartate transaminase.^1–3^ In dogs, the measurement of ALT has been shown to have suboptimal sensitivity and specificity for hepatocellular damage detection.^4,5^ In cats, extrahepatic diseases can further complicate diagnosis by causing elevations in liver enzymes without primary liver disease.^6–8^ A definitive diagnosis typically requires liver biopsy histopathological evaluation, a procedure that carries a morbidity and mortality risk.^9–11^ Consequently, there is a clear need for biomarkers that diagnose hepatic disease more accurately in cats than the current commonly used diagnostic tests.

MicroRNAs (miRNAs) are emerging as potential serum biomarkers for a wide range of diseases in both human and veterinary patients.^12,13^ Their abundance and stability in biological fluids, in conjunction with their relative specificity makes them excellent biomarker candidates.^14–16^ miR-122 is a completely conserved liver-specific miRNA in vertebrates and is the most abundant liver miR.^17–19^ Released into the circulation on hepatic damage, circulating miR-122 has been shown to be a sensitive and specific biomarker for liver injury in humans and dogs.^5,20–22^

In humans, serum miR-122 elevation precedes ALT increase in hepatic injury, making it an earlier biomarker of disease.^21^ Vliegenthart et al^23^ reported in humans with acetaminophen-induced acute liver injury that miR-122 had better specificity than other current biomarkers and better predicted the clinical course of disease than ALT alone.^21,24^ Of the few available miR-122 studies in companion animals, miR-122 has been demonstrated to increase in dogs with liver disease.^20,25,26^ Dirksen et al^25^ found miR-122 to be more sensitive for the detection of hepatocellular injury than ALT. More recently, reference intervals (RIs) for circulating miR-122 in dogs have been generated.^20^

To date, increased miR-122 expression in cats has only been reported in newly diagnosed diabetic cats.^27^ The greater than 40-fold miR-122 increase was presumed to be a result of hepatocyte damage from diabetic ketoacidosis or hepatic lipidosis.^27,28^

The diagnostic utility of miR-122 has not been explored in cats. The aim of this preliminary study was to measure serum concentrations of miR-122 in a cohort of cats with an ALT activity level within the RI (0–83 U/l; ALTn) alongside a cohort of cats with increased ALT activity (>84 U/l; ALTh).

## Materials and methods

### Animals

All cats were enrolled for this study at the Royal (Dick) School of Veterinary Studies (Edinburgh, UK). Cats presented to the Hospital for Small Animals for routine veterinary visits or referral. Cats were only included in the study if ALT measurement was undertaken as part of the clinical investigation. Any surplus serum was then retained for miR-122 measurement. Animals were split into two groups based on ALT measurement: within the RI (ALT 0–83 U/l; ALTn) or greater than the RI (ALT >84 U/l; ALTh). There were 38 cats in the ALTn and 25 cats in the ALTh group. This study was approved by the University of Edinburgh Veterinary Ethics Research Committee (97.21).

### RNA isolation

Serum was stored at −20°C within 4 h of sample collection, then transferred to −80°C for long-term storage.^14,29^ The maximum sample storage time was 380 days (median 222). miRNA was extracted in two batches using a miRNeasy Serum/Plasma kit (Qiagen) following the manufacturer’s guidelines and as per Oosthuyzen et al and Vliegenthart et al.^20,23^ Briefly, total RNA was extracted from 50 μl serum diluted in 150 μl nuclease-free water. RNA was extracted using lysis reagent (1000 μl) and chloroform (200 μl). The RNA was purified on a RNeasy miniElute spin column and eluted in 15 μl RNase-free water and stored at −80°C. Extraction efficiency was assessed by adding 6 × 10^9^ copies/µl of synthetic *Caenorhabditis elegans* miR-39 spike-in control (Norgen Biotek) after the addition of lysis reagent.

### Reverse transcription and real-time PCR

The miScript II Reverse Transcription kit (Qiagen) was used to reverse transcribe cDNA from 2.5 µl RNA according to manufacturer’s guidelines and as per Oosthuyzen et al.^20^ The synthesised cDNA was diluted and 2 µl diluted cDNA template was used in combination with the miScript SYBR Green PCR kit (Qiagen) and specific miScript primer assays for miR-39 and miR-122 (Qiagen). Real-time quantitative PCR (RT-qPCR) was performed in duplicate on the Light Cycler 480 (Roche) at the recommended miScript cycling parameters. The miRNA was quantified as copy per µl by generation of a predetermined standard curve.^20,23^

Repeatability was determined by measuring the intra-assay variability of miR-122 duplicates and expressed as the coefficient of variation (CV%) of concentration (copies/μl), as per the MIQE (minimum information for publication of quantitative real-time PCR) guidelines.^30^ The intra-assay variability (CV%) was deemed acceptable as per guidelines reported in previous studies (CV 8.6%).^31^ Reproducibility was determined by measuring inter-assay variability across two plates and 2 days by measuring miR-122 concentrations (copies/μl) of three reference samples expressed as CV% of concentration (copies/µl) as per MIQE guidelines across two plates (CV 3.5%, 3.6% and 1.9%, respectively).^30^ On each plate a no-enzyme control, omitting the reverse transcriptase enzyme during reverse transcription, and no template control omitting the cDNA in the RT-qPCR, were included in both runs.

### Statistical analysis

Statistical analyses were performed using GraphPad Prism. Mann–Whitney U-test associations were used to assess time in storage until RNA extraction, age and weight differences between groups. Breed and sex differences between groups were assessed by Fisher’s exact test. The difference in miR-122 concentration between the two groups was determined by Mann–Whitney U-test for copies/µl and quantification cycle (Cq) values. The relationship between ALT and miR-122 was assessed by Spearman’s rank correlation and simple linear regression. Cq values <35 were regarded as positive amplification signals and >35 regarded as outside the limit of detection.^31^ *P* values ⩽0.05 were considered to be statistically significant.

## Results

### Cat characteristics

Serum samples from 63 cats were selected for analysis based solely on their ALT values and serum availability. The ALTh group samples were stored at −80°C for longer than those of the ALTn group from time of sampling until RNA extraction (*P* = 0.035). The median ALT activity of the ALTh group was 213 U/l (range 105–1877) and 46 U/l (range 20–78) in the ALTn group.

Clinical characteristics, including signalment and diagnosis, recorded by the case clinicians are summarised in Table 1. The cats in the ALTh group were significantly older (median 13 years) than the ALTn group (median 9.5 years; *P* = 0.0098). For cats with weight data available, those in the ALTn group (n = 30) were significantly heavier (4.94 kg) compared with the ALTh group (n = 24 [3.62 kg]; *P* = 0.013). There was no significant difference in sex distribution between the two groups (*P* = 0.999) and all cats were neutered.

**Table 1:** Signalment and diagnostic features of cats included in this study based on allocated group

	ALTn (n = 38)	ALTh (n = 25)
Median (IQR) age (years)	9.5 (5–12)	13 (3–18)
Median (IQR) body weight (kg)	4.94 (4.1–5.2)	3.62 (3.4–4.5)
Sex		
FN	18	11
MN	20	14
Breed	DSH (n = 23)	DSH (n = 19)
	DLH (nn = 5)	DLH (n = 3)
	Maine Coon (n = 3)	American Shorthair
	British Shorthair (n = 2)	Birman
	Ragdoll (n = 2)	Siamese
	Bengal	
	European Shorthair	
	Exotic Shorthair	
Diagnosis	Cardiomyopathy (n = 7); hyperthyroidism (n = 5); health screen (n = 3 ); dental disease (n = 2); CKD; hypertension; IBD and mammary tumour; Horner’s syndrome; retrobulbar abscess; stress anorexia; thyroid carcinoma; cardiac dysrhythmia; contracted tendons; perioral dermatitis; feline odontoclastic resorptive lesion; mandibular fibrosarcoma; diaphragmatic hernia; intestinal lymphoma; renal lymphoma; FIP; megacolon/atresia ani; pneumothorax and feline allergic airway disease; tracheal stenosis; uveitis; mast cell tumours	Cardiomyopathy (n = 5); hyperthyroidism (n = 2); CKD (n = 2); hyperthyroidism and intestinal mast cell tumour; hyperthyroidism and pancreatitis; cholangiohepatitis and toxoplasmosis; cholangiohepatitis and CKD; epilepsy; lily poisoning; pancreatitis; hepatic mass; cystitis; acute lung injury and diabetes mellitus; cutaneous mast cell tumour; diabetic ketoacidosis; lymphoma; thyrotoxicosis; large cell lymphoma; urolithiasis and extrahepatic biliary tract disorder

Continuous variables are expressed as median and interquartile range (IQR). Where no number is stated, n = 1 ALTn = normal alanine aminotransferase; ALTh = high alanine aminotransferase; FN = female neutered; MN = male neutered; DSH = domestic shorthair; DLH = domestic longhair; BSH = British Shorthair; CKD = chronic kidney disease; IBD = inflammatory bowel disease; FIP = feline infectious peritonitis

### miR-122 serum concentration

**Figure 1:**
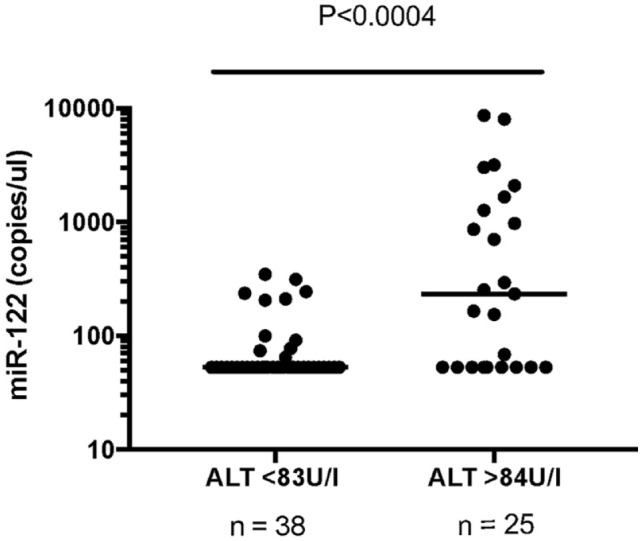
Serum microRNA (miR)-122 concentration in cats with normal or increased alanine aminotransferase (ALT) serum values. Horizontal black line represents the median

**Figure 2:**
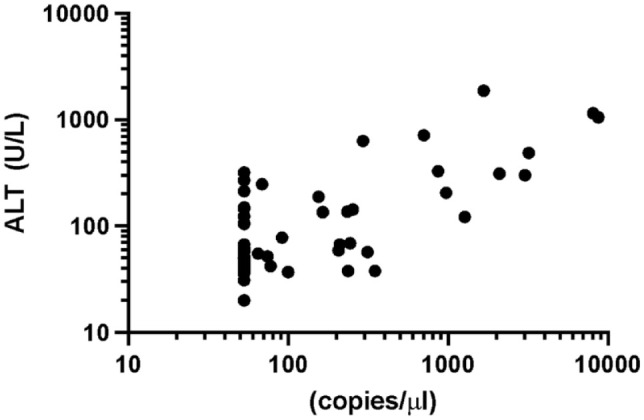
Relationship between serum microRNA-122 concentration and alanine aminotransferase (ALT) activity in 63 cats

miR-122 was significantly higher in the ALTh group than the ALTn group (*P* <0.0004; Figure 1). Median Cq values were also significantly different between groups (*P* = 0.001). The ALTn group median Cq was 35 (range 32.9–35) and the ALTh median Cq was 30.9 (range 26.9–35). In addition, miR-122 concentration had a moderate positive correlation with ALT activity (*P* <0.001; *r* = 0.52 [Figure 2]).^32,33^

## Discussion

This preliminary study demonstrated a significant difference in serum miR-122 concentrations between ALTn cats and ALTh cats. Similarly to our previous observation in dogs, there was also a moderate correlation between increased ALT activity and miR-122 concentration.^20^

The current work was focused on the assessment of feline miR-122 in relation to ALT activity. ALT was selected as the comparator biomarker in this study as it is considered the gold standard marker for hepatocellular damage, being highly sensitive and reasonably specific for hepatocellular injury across species.^34^ Its main limitations are that increased ALT activity can occur with non-hepatic injury, creating false positives, and increased ALT activity does not always correlate with hepatic histopathological findings,^26,34,35^ highlighting the need for new biomarkers for hepatic disease in companion animals.

Histopathological confirmation of hepatobiliary disease was only available for one cat, but of the three cats with a diagnosis of cholangiohepatitis, two had the highest miR-122 values (8780.7 and 8070.5 copies/µl). To make further conclusions on the assessment of miR-122 as a diagnostic test for feline liver disease, informative studies should involve measuring both ALT activity and miR-122 concentration in a greater number of healthy cats, cats with non-hepatic disorders and those with histologically confirmed hepatic disease. This should allow robust miR-122 RIs to be generated, as produced in dogs and humans.^20,36^

miRNAs are known to remain stable when stored for up to 4 years at −20°C or −80°C.^14,37^ Although there was a significant difference in length of time in storage between the two groups from sampling to RNA extraction (*P* = 0.035), the ALTh group was stored for longer than the ALTn group, suggesting that the higher miR-122 concentration in the ALTh group is unlikely to be influenced by differences in storage time. To definitively prove there is no effect of storage on feline miR-122 before use in diagnostic assays, degradation studies comparing fresh and repeat-thawed samples should be conducted, emulating similar work conducted in humans.^14,23,29^

## Conclusions

Our study demonstrates that miR-122 expression is higher in cats with increased ALT activity, indicating that measurement of miR-122 may have diagnostic potential in the assessment of feline hepatic disease. Further studies are needed to examine whether miR-122 has improved sensitivity and specificity compared with currently available diagnostic tests for hepatic disease in cats, and to precisely define its diagnostic utility in this species.
